# CdS sensitized 3D hierarchical TiO_2_/ZnO heterostructure for efficient solar energy conversion

**DOI:** 10.1038/srep05721

**Published:** 2014-07-17

**Authors:** Zhaoke Zheng, Wen Xie, Zhi Shiuh Lim, Lu You, Junling Wang

**Affiliations:** 1 School of Materials Science and Engineering, Nanyang Technological University, 639798, Singapore; 2 The Institute of Scientific and Industrial Research (SANKEN), Osaka University, Mihogaoka 8-1, Ibaraki, Osaka 567-0047, Japan

## Abstract

For conventional dye or quantum dot sensitized solar cells, which are fabricated using mesoporous films, the inefficient electron transport due to defects such as grain boundaries and surface traps is a major drawback. To simultaneously increase the carrier transport efficiency as well as the surface area, optimal-assembling of hierarchical nanostructures is an attractive approach. Here, a three dimensional (3D) hierarchical heterostructure, consisting of CdS sensitized one dimensional (1D) ZnO nanorods deposited on two dimensional (2D) TiO_2_ (001) nanosheet, is prepared via a solution-process method. Such heterstructure exhibits significantly enhanced photoelectric and photocatalytic H_2_ evolution performance compared with CdS sensitized 1D ZnO nanorods/1D TiO_2_ nanorods photoanode, as a result of the more efficient light harvesting over the entire visible light spectrum and the effective electron transport through a highly connected 3D network.

Conversion of solar energy to chemical or electric energy by semiconductor nanomaterials has attracted considerable interest^1^,^2^,^3^. Conventional dye or quantum dot sensitized solar cells are fabricated using mesoporous films which have large surface area^4^. However, a major drawback of the mesoporous nanoparticle photoanode is the inefficient electron transport due to defects such as grain boundaries and surface traps^5^. For instance, the electron diffusion coefficient of TiO_2_ nanoparticles is more than 2 orders of magnitude lower than that of TiO_2_ single crystals^6^. On the other hand, high surface area and efficient electron diffusion are both essential to improve the efficiency of solar energy conversion devices.

Over the past decades, zero-dimensional (0D), one-dimensional (1D) and two-dimensional (2D) nanostructures have attracted much attention due to their unique structure-dependent properties^7^,^8^,^9^. Semiconductors with various tailored morphologies, such as nanoparticles (NPs), nanorods (NRs) and nanosheets (NSs) have been extensively studied for photoelectric applications. Furthermore, heterogeneous nanostructures composed of two or three compounds, usually exhibit superior properties compared with that of a single component^10^,^11^. Therefore, optimal-assembling of multi-component semiconductor nanostructures is an attractive approach for designing next generation solar cells. Up to date, most of the work focused on utilizing one or two kinds of nanostructures to improve the photoelectric conversion performance, such as nanorod/nanoparticle, nanowire/nanosheet et al^12^,^13^,^14^. To simultaneously increase the transport efficiency of carriers as well as the surface area, one can assemble hierarchical 0D, 1D and 2D nanostructures on the electrode.

Here we developed a hierarchical heterostructure with multiple components (2D TiO_2_ NSs, 1D ZnO NRs and 0D CdS NPs) on FTO for efficient solar energy conversion. TiO_2_ and ZnO were chosen in our design due to their low-cost, outstanding chemical stability, and environmentally friendly nature. Both of them are technologically important and have been widely used in the fields of photocatalysis, solar cells, and self-cleaning devices. The integration of ZnO with TiO_2_ will facilitate the photo-generated charge separation and give rise to longer carrier lifetime^15^. More importantly, 2D single-crystal TiO_2_ NSs with reactive (001) facet exhibit superior photocatalytic activity and could also improve the photoelectric conversion performance^16^,^17^,^18^,^19^,^20^. Meanwhile, 1D ZnO NRs can significantly improve the electron transport by providing a direct conduction pathway and reducing the number of interparticle hopings^21^,^22^,^23^,^24^. However, the relatively large band gap of 3.2 eV limits further application of TiO_2_ and ZnO in the visible light region. CdS, with a narrow direct band-gap (2.4 eV) and flat band potential at 0.66 V (pH 7)^25^, can be used as a photosensitizer for the wide band-gap semiconductors TiO_2_ and ZnO. In this work, we synthesize a hierarchical heterostructure, that is, 2D TiO_2_ (001) NSs coated with well-aligned 1D ZnO nanorod arrays, on which 0D CdS NPs are anchored. This is referred to as A-TiO_2_/ZnO/CdS, which combines the advantages of TiO_2_, ZnO and CdS. In this optimized heterostructure, 0D CdS NPs gather the visible light, while 1D ZnO NRs arrays which has a high surface area facilitate sufficient CdS deposition and simultaneously harvest UV-light to generate photoelectrons. The 2D TiO_2_ NSs work as the efficient charge collection and transport path, meanwhile, provide more sites for ZnO deposition. As a reference, a rutile-TiO_2_ NRs/ZnO-NRs/CdS (1D/1D/0D), named as R-TiO_2_/ZnO/CdS, is also fabricated and the influence of different morphologies of the heterogeneous architectures on photoelectric performance is systematically studied.

## Results

### Morphology and key processes of A-TiO_2_/ZnO/CdS

**Figure 1:**
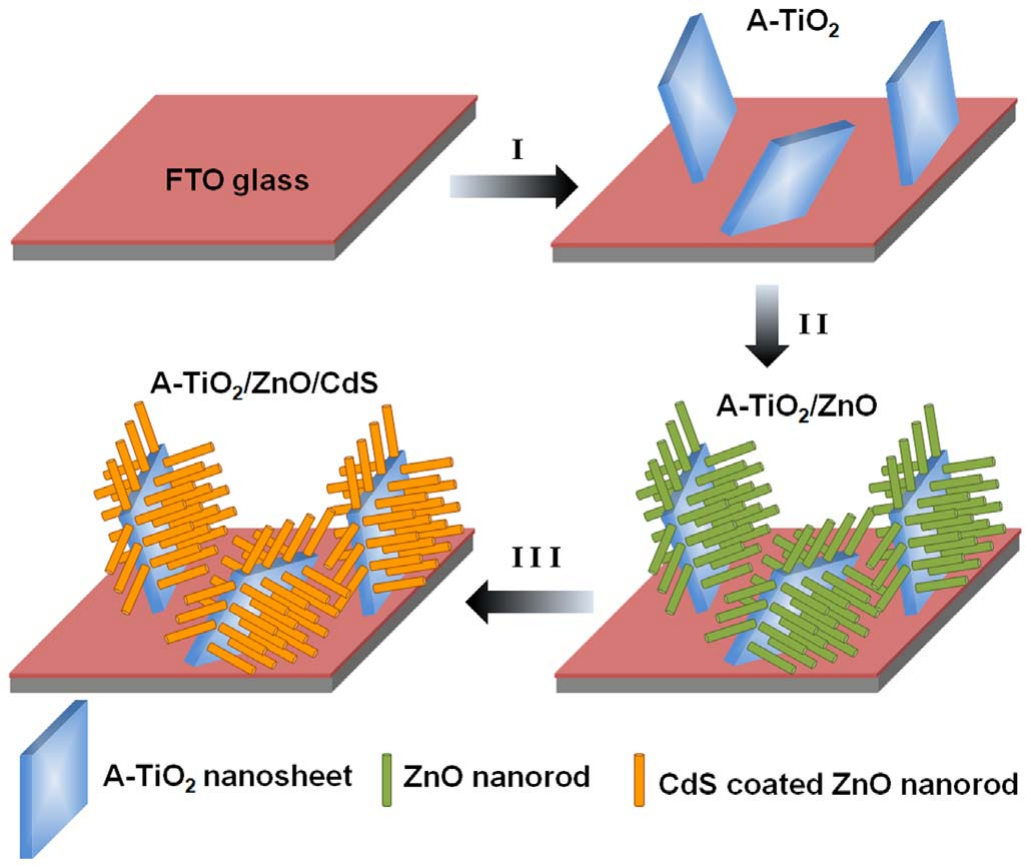
Schematic illustration of the fabrication process of CdS sensitized A-TiO_2_/ZnO heterostructure on FTO glass substrate.

The fabrication process of CdS sensitized A-TiO_2_/ZnO heterostructure on FTO glass substrate is shown in Figure 1. The 2D single-crystal TiO_2_ NSs were grown on FTO by a one-step hydrothermal method^26^. The ZnO NRs arrays were deposited on the surface of TiO_2_ via a seed-mediated method^27^, while the seed layer was pre-deposited by Pulsed Laser Deposition (PLD). After that, CdS was deposited on A-TiO_2_/ZnO by successive ion-layer adsorption and reaction (SILAR) technique^28^, which is based on the successive surface adsorption of ions and thus proceeds via a layer-by-layer buildup of the film^29^.

**Figure 2:**
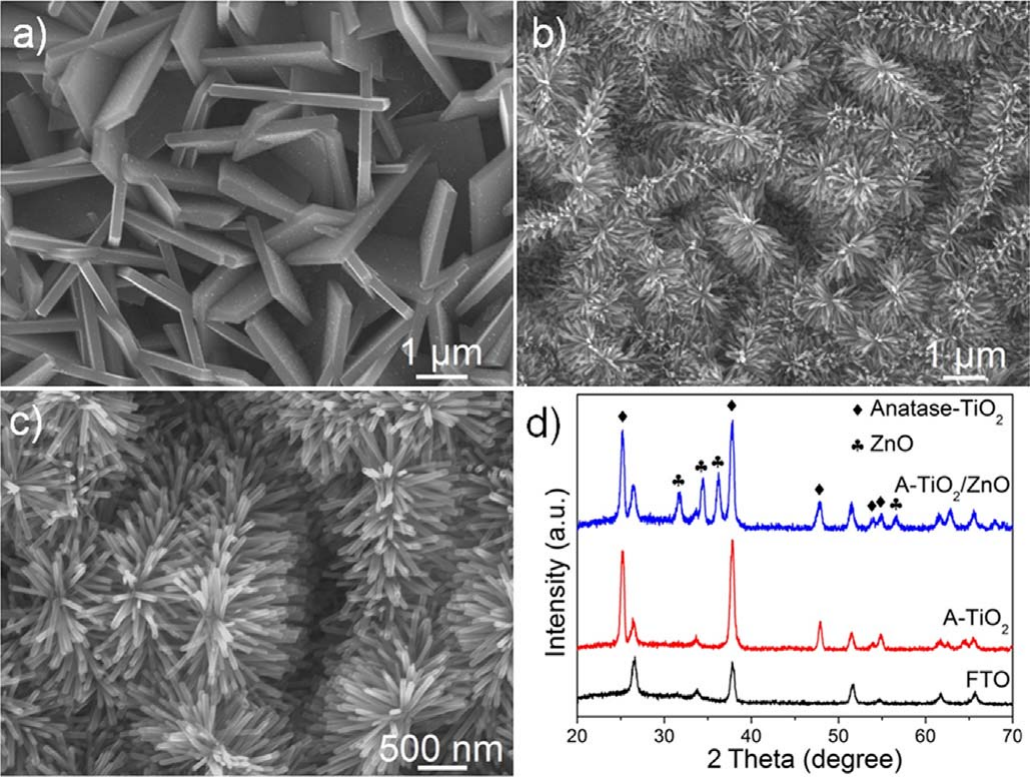
SEM images and XRD patterns of the nanostructures obtained during the growth of hierarchical TiO_2_/ZnO heterostructure on FTO. (a) Growth of anatase TiO_2_ (001) tetragonal nanosheet arrays on FTO (A-TiO_2_), (b, c) Deposition of ZnO nanorod arrays on TiO_2_ (A-TiO_2_/ZnO). (d) XRD patterns of FTO, A-TiO_2_ and A-TiO_2_/ZnO.

As shown in Figure 2a, the single-crystalline TiO_2_ (001) NSs, with side length of ~2 μm and thickness of ~100 nm, grow vertically or obliquely on the FTO substrate (Figure S1). The XRD pattern (Figure 2d) shows that the products are pure anatase phase of TiO_2_ with lattice constant *a* = 3.7852 Å (JCPDS No. 21-1272). Compared with the powder diffraction pattern, the (004) diffraction peak is significantly enhanced, indicating the exposing of large percentage of {001} facet. After the deposition of ZnO seed layer by PLD, well-aligned ZnO NRs-arrays with average diameter of 60 nm and length of 800 nm were vertically grown on the TiO_2_ {001} and {101} facets, respectively (Figure S2). Notably, the low-magnification SEM images (Figure S2a) show that the A-TiO_2_/ZnO heterostructure retain the original morphology of TiO_2_ NSs. Such kind of well-aligned ZnO arrays will facilitate efficient photogenerated-electron transfer from ZnO to TiO_2_ surface. The XRD pattern (Figure 2d) shows that all the diffraction peaks of ZnO nanorods can be indexed to a pure wurtzite hexagonal phase of ZnO.

**Figure 3:**
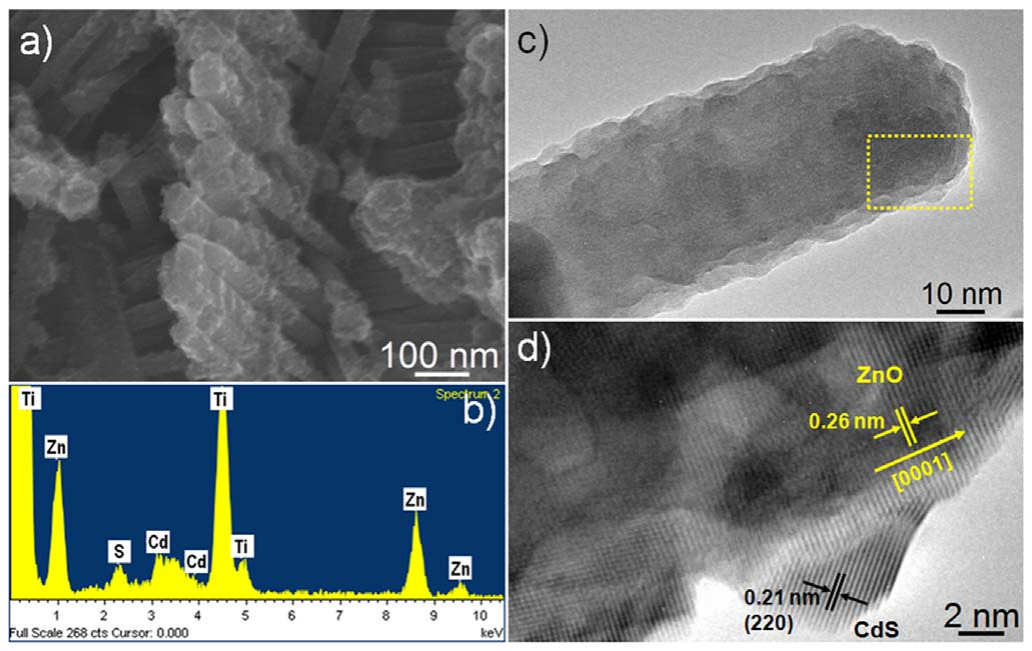
Morphological characterization of A-TiO_2_/ZnO/CdS. (a) SEM images and (b) EDS spectra of CdS sensitized A-TiO_2_/ZnO heterostructure (A-TiO_2_/ZnO/CdS). (c) TEM and (d) HRTEM images of a single CdS/ZnO nanorod.

Figure 3a shows a typical SEM image of ZnO NRs arrays after the deposition of CdS *via* SILAR process. It can be found that the surface of ZnO NRs is homogeneously covered by a thin layer of CdS. The coating of CdS NPs on ZnO NRs as shown in Figure 3a is further characterized with energy dispersive X-ray spectroscopy (EDS). The EDS spectrum in Figure 3b reveals that the nanocomposite consists of zinc, cadmium, and sulphur. The titanium can also be detected as the substrate of ZnO NRs arrays.

The ZnO/CdS core-shell structure is further demonstrated with transmission electron microscopy (TEM) images in Figure 3c, which show evidence of morphological changes of the ZnO NRs upon CdS deposition, such as significant surface roughening and diameter increase. The single-crystalline core (Figure 3d) shows a fringe spacing of 0.26 nm, which matches well with the *d*-spacing of the (0002) plane of hexagonal ZnO, confirming that the ZnO nanorods are preferentially oriented in the c-axis direction. A lattice fringe spacing of 0.21 nm in the crystallites matches well with the interplanar spacing of the (220) plane of cubic CdS, which demonstrates that the CdS NPs deposited on the surface of ZnO NRs are well-crystallized and the interface between them is of high quality. This will facilitate the photo-generated electron transfer from CdS to ZnO. The XRD pattern of A-TiO_2_/ZnO/CdS (Figure S3d) does not show the diffraction peak of cubic CdS even though they are well-crystallized. One possible reason is the overlap between the (111) diffraction peak of CdS and the (110) diffraction peak of SnO_2_ (FTO). Another reason is the amount and particle size of CdS NPs is too small to be detected.

### Synthesis and characterization of R-TiO_2_/ZnO/CdS

To systematically study the influence of different morphologies of the heterogeneous architectures on the photoelectric performance, a reference sample was fabricated and characterized. Figure S4 shows the fabrication process of CdS sensitized R-TiO_2_ NRs/ZnO NRs heterostructure (R-TiO_2_/ZnO/CdS) on FTO glass substrate, which is similar to that of A-TiO_2_/ZnO/CdS, except that rutile TiO_2_ NRs arrays (R-TiO_2_) are grown on FTO^30^ at the beginning instead of A-TiO_2_ NSs arrays.

Figure S5 shows typical SEM images of the R-TiO_2_ NRs arrays grown on FTO surface. The images at different magnifications reveal that the entire surface of the FTO substrate is uniformly covered with TiO_2_ NRs. Tetragonal NRs with square top facets are nearly perpendicular to the FTO substrate, and the average diameter is 90 nm. The XRD patterns of the sample show that all the diffraction peaks match well with the tetragonal rutile phase. Absence of some diffraction peaks that are normally present in polycrystalline samples demonstrates that the nanorods are not only aligned but are also single crystalline throughout their length. After the deposition of ZnO seed layer by PLD, ZnO NRs-arrays with average diameter of ~240 nm were vertically grown on TiO_2_ NRs film (Figure S6). The XRD pattern (Figure S6d) clearly shows the diffraction peaks of wurtzite ZnO and rutile TiO_2_.

Figure S7 shows the SEM images of R-TiO_2_/ZnO/CdS heterostructure obtained with different SILAR deposition cycles. The CdS layer deposited on top of the R-TiO_2_/ZnO after 25 SILAR cycles exhibits a flat surface, which is due to the relative smaller surface area of R-TiO_2_/ZnO (top region for CdS deposition) compared with A-TiO_2_/ZnO substrate. Further increase the SILAR deposition to 50 cycles leads to cluster growth and echinus-like CdS microstructures. Thus, for R-TiO_2_/ZnO/CdS, the interface area between ZnO and CdS is much smaller compared with A-TiO_2_/ZnO/CdS heterostructure, while the latter will offer efficient charge transfer between CdS and ZnO. Furthermore, the large exposed surface area of A-TiO_2_/ZnO/CdS will generate efficient charge transfer between the electrolyte and the photoanodes.

## Discussion

**Figure 4:**
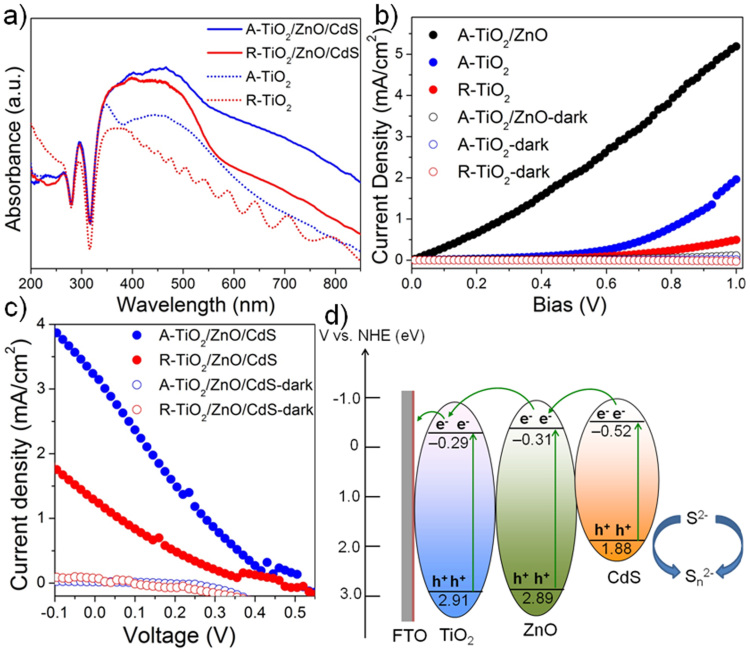
Photoelectric properties of hierarchical nanostructures on FTO substrates. (a) UV–Vis diffuse reflectance spectra of the samples. (b) *J*–*V* curves of R-TiO_2_, A-TiO_2_, A-TiO_2_/ZnO in darkness and under illumination. (c) *I*−*V* characteristics of A-TiO_2_/ZnO/CdS and R-TiO_2_/ZnO/CdS solar cells in darkness and under illumination. (d) Schematic illustration of band energy positions and the charge transfer process of the TiO_2_/ZnO/CdS heterostructure.

UV-vis spectra of the A-TiO_2_/ZnO/CdS, the R-TiO_2_/ZnO/CdS, and the arrays of bare TiO_2_ on FTO substrates were characterized (Figure 4a). Compared with the R-TiO_2_ NRs arrays (red dotted line), the A-TiO_2_ (001) NSs arrays have a stronger absorption in the UV–Vis region (blue dotted line), which is due to its multiple reflection structure of the NSs-arrays. To eliminate the influence of film thickness on the light harvesting efficiency, the cross-sectional morphology was characterized (Figure S8) and the result shows that the film thicknesses of these two different structures are nearly the same. The hierarchical A-TiO_2_/ZnO/CdS also exhibit a significantly improved absorption in the visible region compared with the R-TiO_2_/ZnO/CdS, therefore, are expected to provide a more efficient light harvesting and an enhanced photoelectric performance.

Figure 4b shows the *J*–*V* curves of R-TiO_2_, A-TiO_2_, A-TiO_2_/ZnO in dark and under illumination. The A-TiO_2_ NSs arrays exhibits a significantly enhanced (3.4 times in photocurrent at 0.8 V) photoelectric response compared with the R-TiO_2_, which is due to its highly reactive (001) facet and good crystallinity. When combined with ZnO NRs arrays, the photocurrent density at 0.8 V is about 4.9 times that of the bare A-TiO_2_, indicating that the TiO_2_/ZnO heterojunction can facilitate the photo-generated charge separation and hence improve the photoelectric performance. With Pt counter-electrodes and polysulfide electrolyte, we obtained a simple solar cell and characterized the photoelectric performance (Figure 4c). When using the A-TiO_2_/ZnO/CdS as the photoanode, a short-circuit photocurrent (*J*_sc_) of 3.24 mA/cm^2^ is observed, which is much higher than that of the R-TiO_2_/ZnO/CdS photoanode (1.30 mA/cm^2^). The cell with A-TiO_2_/ZnO/CdS as the photoanode shows a fill factor (FF) and efficiency of 0.17 and 0.51%, respectively, which exhibit a significant increase when compared with that of the R-TiO_2_/ZnO/CdS photoanode (0.11 and 0.13%). The improved interfacial charge transfer, efficient photo-generated electron collection from the 0D to 2D nanostructures, as well as increased light absorption are the main reasons for the enhanced performance.

Moreover, band position calculations (see detail in supporting information) suggest that TiO_2_, ZnO and CdS have the staggered Z-scheme energy potentials that can reduce the recombination of the photogenerated carriers within the three semiconductors. Under solar light irradiation, the 0D CdS NPs are excited and the as-photoinduced electrons migrate to the 1D ZnO, which could be subsequently collected by the 2D TiO_2_ NSs and finally migrate to the FTO substrate. Meanwhile, the redox electrolyte (sulfide/polysulfide) scavenges the holes and thus ensures regeneration of the CdS.

To further investigate the role of each component, the photoelectric performance was characterized under different light irradiation. As shown in Figure S9, under visible light irradiation, the sample still shows photoelectric response, which is arises from the CdS NPs. Under the full spectrum light irradiation, the photocurrent density is higher than that under UV light, indicating that such a hierarchical composite could utilize both UV and visible band of solar light.

**Figure 5:**
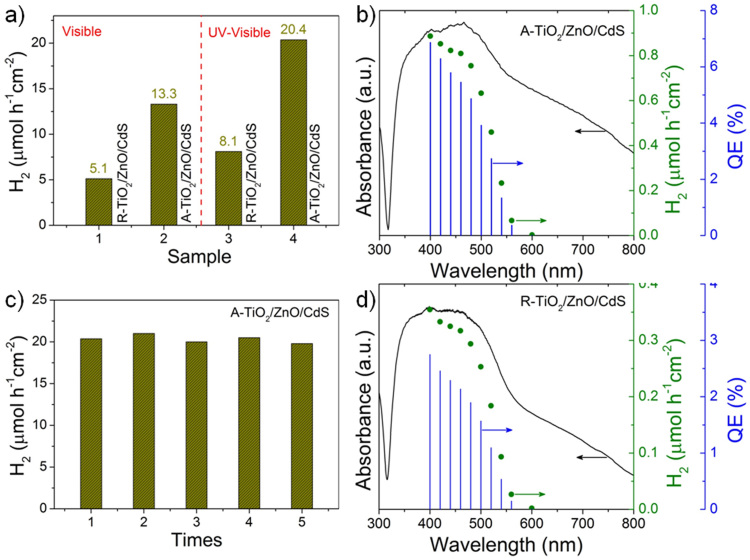
Photocatalytic H_2_ evolution. (a) H_2_ evolution rate of A-TiO_2_/ZnO/CdS and R-TiO_2_/ZnO/CdS film under visible (λ > 420 nm) and UV-visible light irradiation. (c) H_2_ evolution over A-TiO_2_/ZnO/CdS during repeated photocatalytic experiments under UV-visible light irradiation. Action spectra of H_2_ evolution and quantum efficiency (QE) over (b) A-TiO_2_/ZnO/CdS and (d) R-TiO_2_/ZnO/CdS.

Photocatalytic H_2_ production from water using semiconductor photocatalysts is an attractive process for solving energy and environmental problems. As shown in Figure 5a, the hierarchical heterostructures exhibit efficient H_2_ evolution. Under visible light irradiation, the H_2_ evolution rate of A-TiO_2_/ZnO/CdS is 13.3 μmol h^−1^cm^−2^, which is 2.6 times that of R-TiO_2_/ZnO/CdS. The higher activity under UV-visible light irradiation is due to the excitation of all the three components as well as enhanced charge separation. Figure 5c displays the stability of A-TiO_2_/ZnO/CdS for H_2_ evolution under UV-visible light irradiation. There is no obvious activity decrease after each run, indicating the good stability of our sample. Furthermore, the action spectra were obtained using monochromatic light at intensity of 4.3 mW cm^−2^. The quantum efficiency (QE) at each centered wavelength of the monochromatic light was calculated from the ratio of twice the number of H_2_ molecules to the number of incident photons using the following equation: QE = (2 × the number of H_2_ molecules/the number of incident photons) × 100%. Figure 5b and 5d show the action spectra of H_2_ evolution and quantum efficiencies. For both samples, the H_2_ evolution rate and quantum efficiencies decreased obviously at around 520 nm with band-pass filters, revealing that the visible-light response for H_2_ evolution is due to the band-gap transition of CdS^31^. The QE of H_2_ evolution for A-TiO_2_/ZnO/CdS obtained using a 460-nm band-pass filter was still as high as 5.5%, which is much higher than that of R-TiO_2_/ZnO/CdS (2.1%).

In summary, we have developed a well-aligned, structurally-optimized heterostructure with multiple components (2D TiO_2_ NSs, 1D ZnO NRs and 0D CdS NPs) for efficient solar energy conversion. The structure−property relationship is systematically studied by comparing with a reference photoanode, R-TiO_2_ NRs/ZnO NRs/CdS NPs. The designed A-TiO_2_/ZnO/CdS heterostructure shows a significantly enhanced photoelectric performance and H_2_ evolution activity as a result of improved light absorption and efficient electrons transport through a highly connected 3D network. We believe that this strategic design of 3D architectures from 2D to 0D nano-components can be further extended to the synthesis of various heterostructures with promising applications in clean energy, optoelectronics, sensing and so on.

## Methods

### Materials

Tetrabutyl titanate (97%), ammonium hexafluorotitanate ((NH_4_)_2_TiF_6_), zinc nitrate hexahydrate (Zn(NO_3_)_2_·6H_2_O), hexamethylenetetramine (HMTA C_6_H_12_N_4_), cadmium nitrate tetrahydrate (Cd(NO_3_)_2_·4H_2_O, 98%), sodium sulfide hydrate (Na_2_S·9H_2_O) were purchased from Sigma Aldrich. All the chemicals were used as received without further purification.

### Substrate Preparation

Fluorine-doped tin oxide (FTO) glasses (1 cm × 2 cm) were cleaned ultrasonically with acetone and ethanol for 10 min, respectively, and then dried with nitrogen before TiO_2_ growth.

### Growth of TiO_2_ Arrays on FTO Substrate

(1) Anatase TiO_2_ (001) tetragonal nanosheet arrays (A-TiO_2_) were synthesized with an improved method similar to that described by Yang^26^. In a typical procedure, 1 mL of tetrabutyl titanate was mixed with 60 mL hydrochloric acid (5 M) under vigorous stirring for 10 min, then 0.5 g (NH_4_)_2_TiF_6_ was added into the solution and stirred for another 10 min. The resulting solution was transferred in a dried Teflon autoclave. After that, two pieces FTO glasses were placed at the bottom of the Teflon autoclave with the conductive side facing up. The Teflon autoclave was kept at 180 °C for 16 h. After cooling down to room temperature, the FTO substrates were taken out and rinsed with deionized water thoroughly and then dried at 60 °C. (2) Rutile TiO_2_ nanorod arrays (R-TiO_2_) were synthesized following similar procedure except (NH_4_)_2_TiF_6_ was removed, and the FTO substrates were placed at an angle against the wall of the Teflon autoclave with the conducting side facing down. The hydrothermal synthesis was conducted at 150°C for 6 h.

### Growth of ZnO Nanorod-Arrays on TiO_2_ Arrays

ZnO nanorod-arrays were synthesized by a seed-mediated method^27^. First, ZnO seed layer was deposited on the surface of TiO_2_ arrays by Pulsed Laser Deposition (PLD). The distance between the ZnO target and the substrate was ~50 mm. The vacuum chamber was evacuated to a base pressure of 1 × 10^−5^ Pa and then retained at an oxygen partial pressure of 3 Pa. The energy at the target surface and repetition rate of the laser was 52 mJ and 5 Hz, respectively. Hydrothermal ZnO growth was carried out by suspending the above-mentioned substrates upside-down in an aqueous solution containing 25 mM zinc nitrate hexahydrate and 25 mM hexamethylenetetramine at 90 °C. The typical growth time is 4 hours. The samples were then removed from solution, rinsed with deionized water, and dried at 50 °C. The ZnO nanorod-arrays modified TiO_2_ films were mentioned as A-TiO_2_/ZnO and R-TiO_2_/ZnO, respectively.

### CdS Deposition

CdS was deposited on the A-TiO_2_/ZnO and R-TiO_2_/ZnO samples by successive ion-layer adsorption and reaction (SILAR) technique^28^. Typically, the TiO_2_/ZnO films were successively immersed in two different aqueous solutions for 20 s each, first in 50 mM Cd(NO_3_)_2_ in methanol and then in 50 mM Na_2_S in methanol/water (1:1/v:v). Between each immersion step, the samples were rinsed with methanol for 20 s to remove excess ions that were weakly bound to the surfaces. This immersion cycle was repeated for up to 50 cycles and, after finishing the coating, the samples were dried at 40 °C. The as-prepared samples were named as A-TiO_2_/ZnO/CdS and R-TiO_2_/ZnO/CdS, respectively.

### Fabrication of solar cells

Platinum (Pt) coated silicon wafers counter-electrodes were prepared by PLD. Solar cells were prepared by assembling and bonding the TiO_2_/ZnO/CdS electrodes with the Pt counter-electrodes. The two electrodes were separated by a 60 μm thick polypropylene spacer, and the internal space of the cell was filled with a polysulfide electrolyte (1.0 M S, 1.0 M Na_2_S and 0.1 M NaOH in deionized water).

### Characterization

X-ray diffraction patterns were obtained using a Shimadzu XRD-6000 X-ray diffractometer with Cu Kα radiation (λ = 1.5418 Å). Field emission scanning electron microscopy images were obtained using a JSM-7600F microscope. The EDS was taken on an OXFORD INCA X-Max Energy Dispersive X-Ray Spectroscopy. High-resolution transmission electron microscopy (HRTEM) measurements were carried out on JEM-2010 operated at 200 KV. *J*–*V* measurements were performed using a Keithley 2400 SourceMeter with a custom-made LabTracer program. A 300 W Xenon Arc Lamp (Newport) with appropriate filter was used as the light source.

### Photocatalytic Hydrogen Production Activity Tests

Typically, the TiO_2_/ZnO/CdS electrode (0.3 cm^2^) was immersed in 4 mL of aqueous solution containing 0.1 M Na_2_S and 0.1 M Na_2_SO_3_ bubbled with Ar, and sealed with a rubber septum. A 300 W Xenon Arc Lamp (Asahi Spectra, LAX-C100) with appropriate filter was used as the light source to provide simulated sunlight (AM 1.5G). For visible-light hydrogen production, a 420 nm cutoff filter was used to remove UV light. The amount of H_2_ in the gas phase was measured using a Shimadzu GC-8A gas chromatograph equipped with an MS-5A column and a thermal conductivity detector (TCD). To obtain an action spectrum in this reaction system, photocatalytic H_2_ formation was carried out under irradiation of monochromated light from a Xe lamp (Asahi Spectra; 4.3 mW cm^−2^) with band-pass filters (light width: ±5 nm).

## Author Contributions

J.W. proposed and guided the project. Z.Z. designed and preformed the experiments. W.X. characterized the cross-section morphologies and measured the action spectra. Z.L. and L.Y. performed the PLD deposition. Z.Z. wrote the paper. J.W. revised the manuscript. All authors analyzed and discussed the experimental results.

## Supplementary Material

Supplementary Information Supplementary information
