# Supplementary material for: CdS sensitized 3D hierarchical TiO2/ZnO heterostructure for efficient solar energy conversion
Source: Sci Rep. 2014 Jul 17;4:5721. doi: 10.1038/srep05721 (PMC4101473; doi:10.1038/srep05721)
Supplement: Supplementary Information [file srep05721-s1.pdf]

Supplementary information

CdS Sensitized 3D Hierarchical TiO_2/ZnO Heterostructure for Efficient Solar Energy Conversion

Zhaoke Zheng[1], Wen Xie[2], Zhi Shiuh Lim[1], Lu You[1] & Junling Wang[1]*

[1]School of Materials Science and Engineering, Nanyang Technological University, 639798, Singapore, [2]The Institute of Scientific and Industrial Research (SANKEN), Osaka University, Mihogaoka 8-1, Ibaraki, Osaka 567-0047, Japan.

* Corresponding author, E-mail: jlwang@ntu.edu.sg

Band position calculations

For an inorganic semiconductor, the valence band (VB) and conduction band (CB) position at the point of zero charge can be calculated by the following empirical formulas with respect to the normal hydrogen electrode (NHE)[1]:

$$E_{VB} = \chi - E^{e} + 0.5E_{g} \quad (1)$$

$$E_{CB} = \chi - E^{e} - 0.5E_{g} \quad (2)$$

where χ is the absolute electronegativity of the semiconductor, which is defined as the geometric mean of the absolute electronegativity of the constituent atoms, E_e is the energy of free electrons on the hydrogen scale (*ca.* 4.5 eV), E_{VB} is the VB edge potential, E_{CB} is the CB edge potential

and E_g is the band gap of the semiconductor. The calculated band positions of TiO_2, ZnO and CdS are summarized in **Table S1**.

Table S1. Band energy positions of TiO_2, ZnO and CdS.

semiconductors	χ (eV)	E_g (eV)	E_{VB} (eV) *vs.* NHE	E_{CB} (eV) *vs.* NHE	E_{VB} (eV) *vs.* AVS	E_{CB} (eV) *vs.* AVS
TiO_2	5.81	3.2	2.91	–0.29	–7.41	–4.21
ZnO	5.79	3.2	2.89	–0.31	–7.39	–4.19
CdS	5.18	2.4	1.88	–0.52	–6.38	–3.98

Notes: The energy positions of band edges with respect to the absolute vacuum scale (AVS) can be calculated using: $E_{(AVS)} = -E_{(NHE)} - 4.50$.

Reference

1. Y. Xu, and M. A. A. Schoonen, *Am. Mineral.*, 2000, **85**, 543.

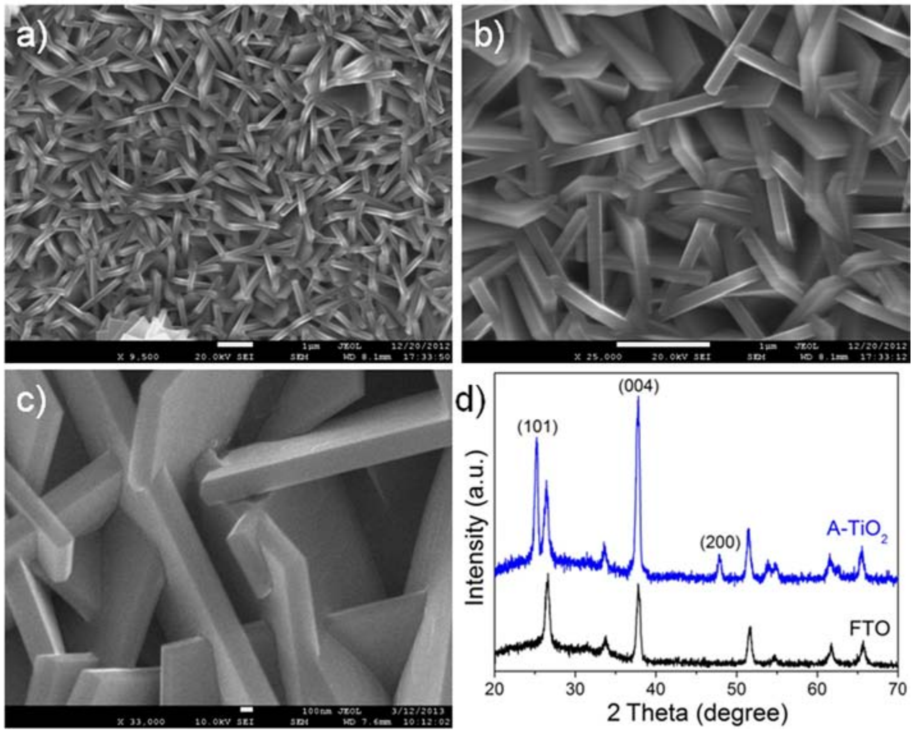

Figure S1. (a–c) SEM images of anatase TiO_2 (001) tetragonal nanosheet arrays grown on FTO (A-TiO_2). (d) XRD patterns of FTO and A-TiO_2.

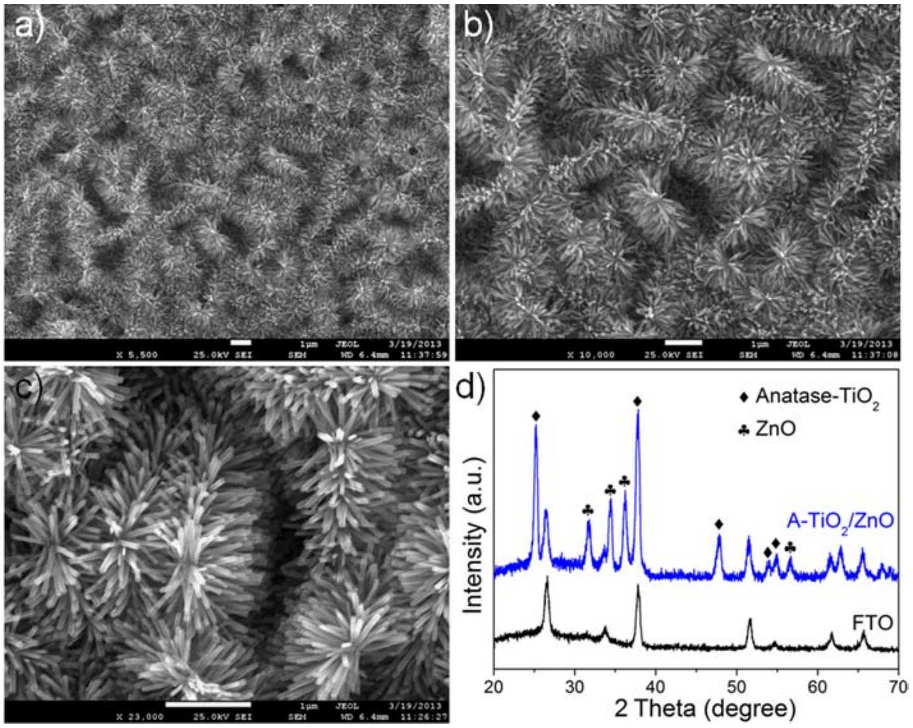

Figure S2. (a–c) SEM images of ZnO nanorod arrays on anatase TiO_2 (001) tetragonal nanosheet (A-TiO_2/ZnO). (d) XRD patterns of FTO and A-TiO_2/ZnO.

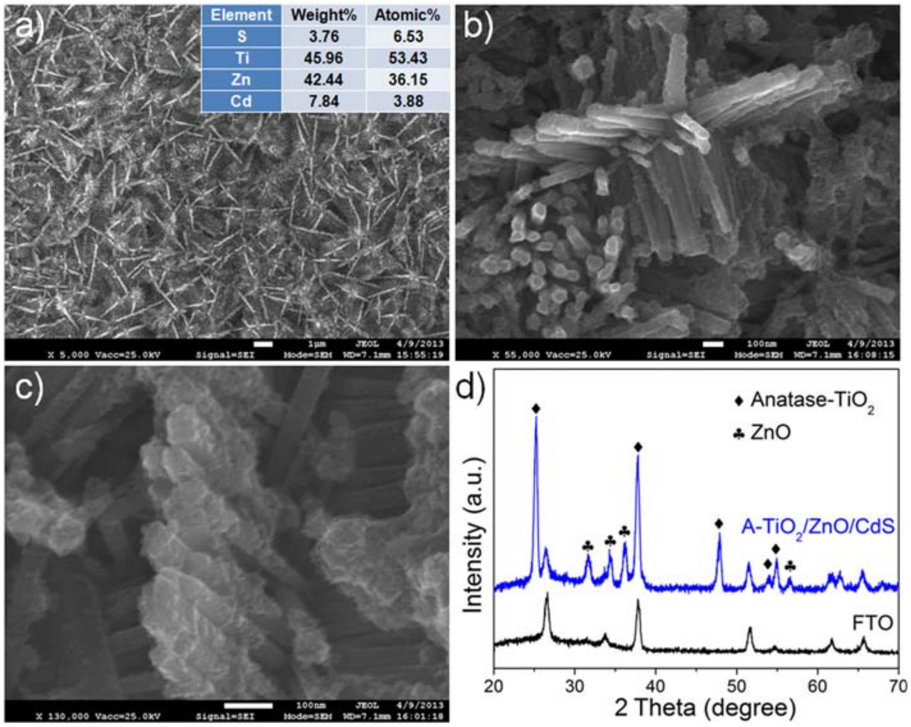

Figure S3. (a–c) SEM images of CdS sensitized A-TiO_2/ZnO heterostructures (A-TiO_2/ZnO/CdS). The inset shows the element contents obtained by EDS. (d) XRD patterns of FTO and A-TiO_2/ZnO/CdS.

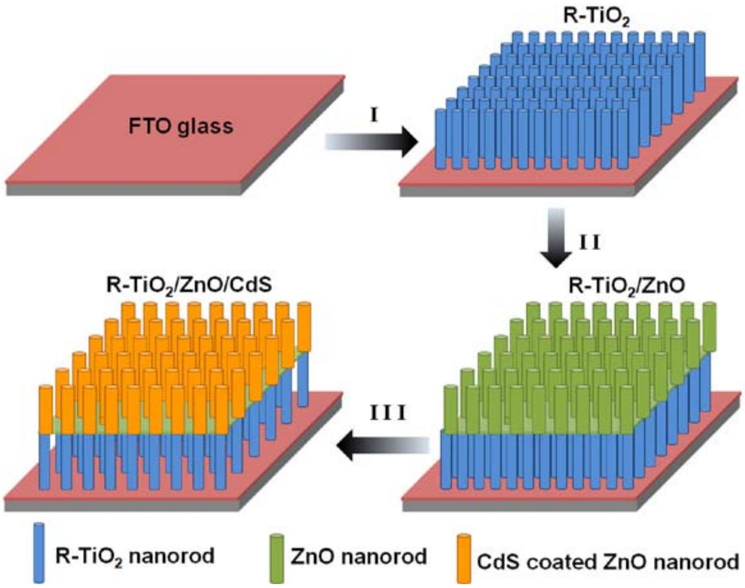

Figure S4. Schematic illustration of the fabrication process of CdS sensitized R-TiO_2/ZnO heterostructures on FTO glass substrate.

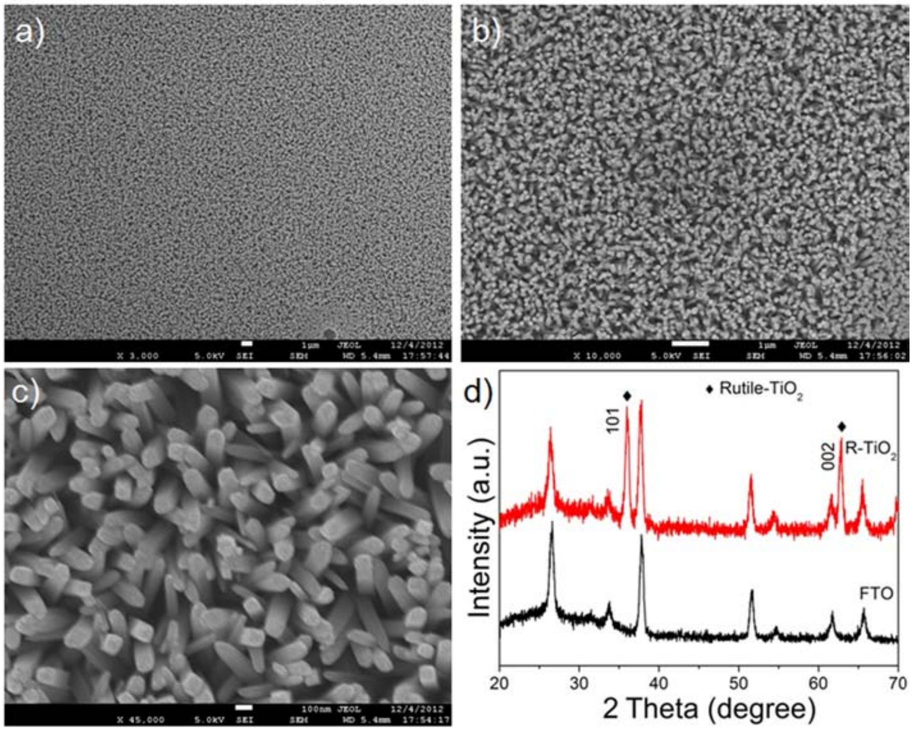

Figure S5. (a–c) SEM images of rutile TiO_2 nanorod arrays grown on FTO (R-TiO_2). (d) XRD patterns of FTO and R-TiO_2.

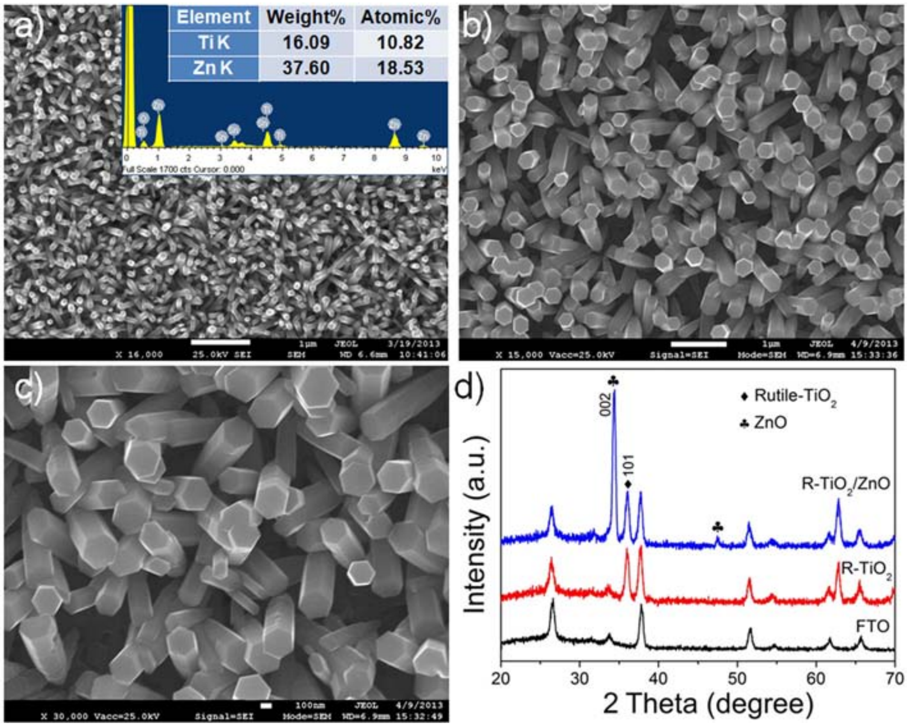

Figure S6. (a–c) SEM images of ZnO nanorod arrays grown on rutile TiO_2 nanorod arrays (R-TiO_2/ZnO). (d) XRD patterns of FTO and R-TiO_2/ZnO.

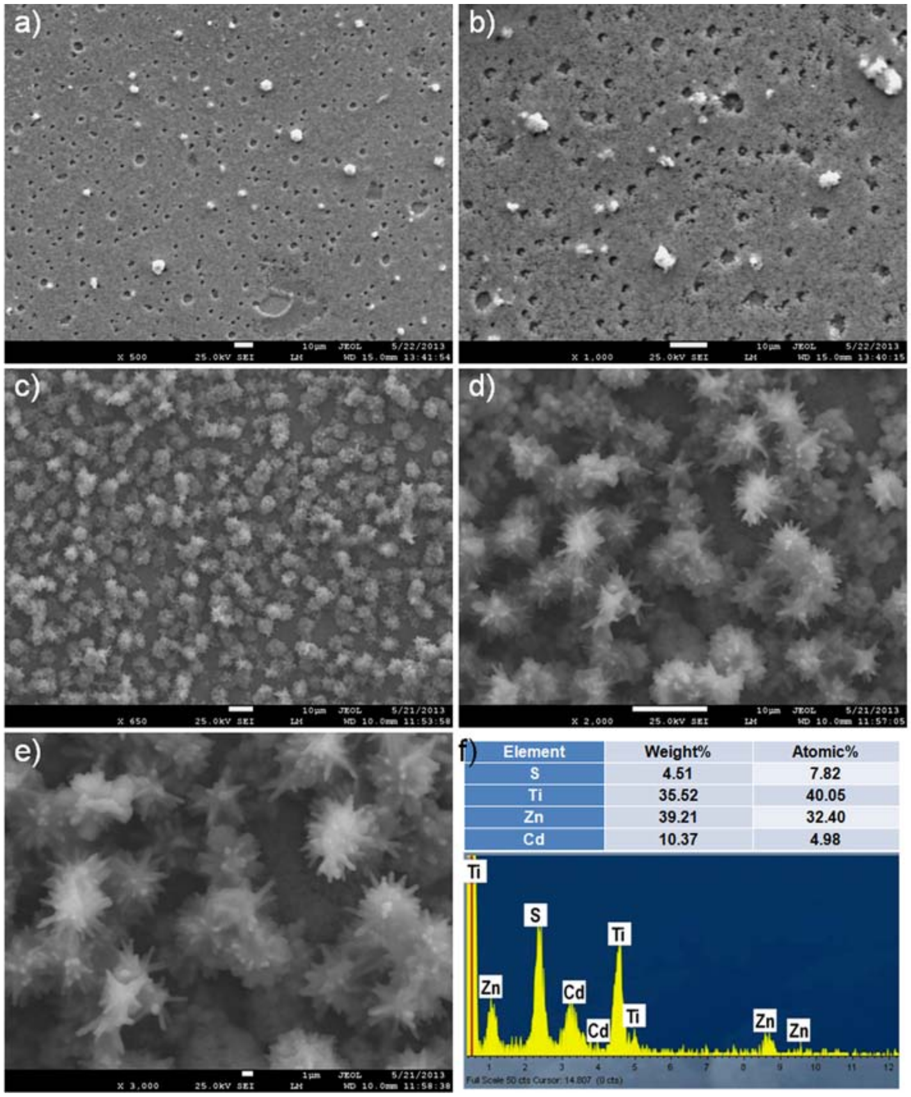

Figure S7. SEM images of CdS sensitized R-TiO_2/ZnO heterostructures (R-TiO_2/ZnO/CdS) ontained by different SILAR deposition cycles. (a,b) 25 cycles, (c–e) 50 cycles. (f) EDS spectra of R-TiO_2/ZnO/CdS.

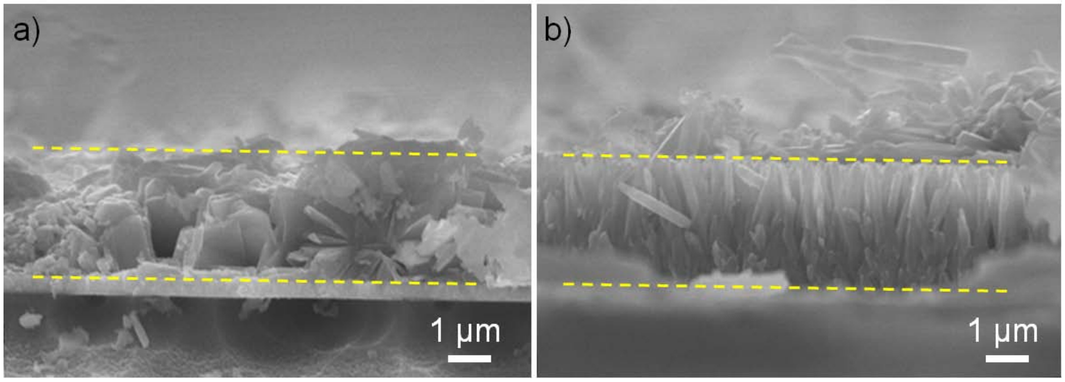

Figure S8. Cross-sectional SEM images of (a) A-TiO_2 (001) NSs arrays and (b) R-TiO_2 NRs arrays.

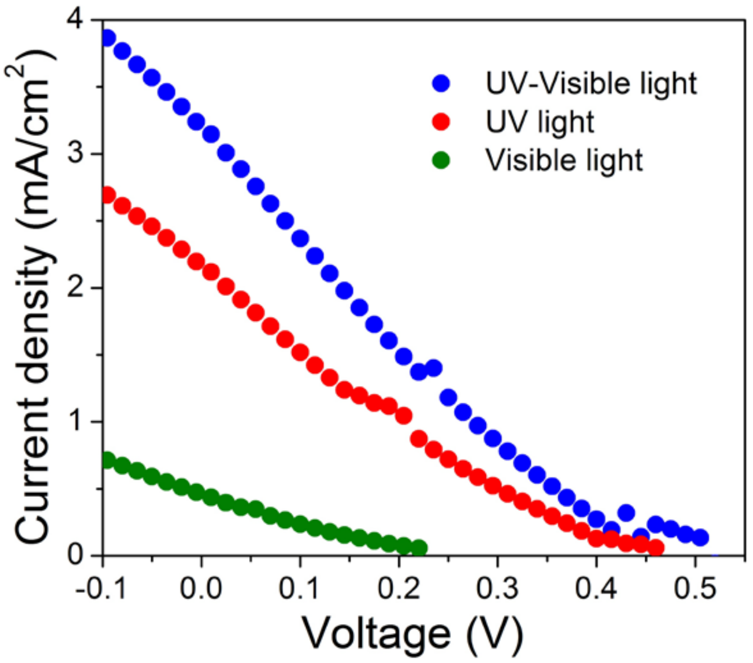

Figure S9. *I*–*V* characteristics of A-TiO_2/ZnO/CdS solar cells under different light illumination. UV light: $\lambda < 420$ nm; Visible light: $\lambda > 420$ nm.
